# Sleep quality depends not only on radicular pain but also on other factors

**DOI:** 10.1590/1806-9282.20231367

**Published:** 2024-05-03

**Affiliations:** 1 Universidade Federal de São Paulo, Paulista School of Medicine, Discipline of Neuroscience – São Paulo (SP), Brazil.; 2 Universidade Federal de São Paulo, Paulista School of Medicine, Center for Neuroscience and Women's Health “Professor Geraldo Rodrigues de Lima” – São Paulo (SP), Brazil.; 3 Neurology and Neurophysiology Center, Department of Neurology – Vienna, Austria.

Dear Editor,

We read with interest the article by Ay and Tuna, which is a case-control study involving 42 patients with lumbar radicular pain due to a herniated disc and its influence on sleep quality and lower limb functionality^1^. All patients underwent needle electromyography, visual analog scale (VAS) test, Pittsburgh Sleep Quality Index (PSQI), and lower extremity functional scale (LEFS) assessment to identify the nerve roots that were affected^1^. It was found that radiculopathy did not affect sleep quality and lower extremity functionality but it was hypothesized that the degree of disc herniation might influence the two outcome measures. The study is excellent but it raises concerns that should be discussed.

The first limitation of the study is that the current pain medications such as orthopedic therapy and physical therapy were not included in the analysis. Since the suppression of pain depends largely on the type and intensity of treatment and pain greatly affects sleep quality, it is important to know all types of treatments that the patients have received for their radicular pain before inclusion. How many patients received hives therapy, infiltration, or CT-guided infiltration in addition to oral or intravenous pain killers before inclusion? It is also important to know the details of medications that were regularly taken by patients in addition to painkillers.

The second limitation is that the inclusion and exclusion criteria were not well defined. Previous spinal surgery, radiculitis due to Borreliosis or Elsberg syndrome, varicositas spinalis, myelitis, vertebrostenosis, subdural hematoma, and syringomyelia were not mentioned as exclusion criteria. Did any of the patients undergo previous spinal surgery before inclusion?

The third limitation is that the included patients did not undergo assessment of depression and anxiety. As depression can result from chronic pain and can strongly influence pain perception and processing, it is crucial to know the number of the included patients who also suffered from depression in addition to their radicular pain. People with depression may aggravate their pain syndrome compared to people without depression.

The fourth limitation is that, in addition to pain, factors that determine sleep quality and intensity were not included in the assessment. We should know the number of patients who had alcohol addiction, regularly took tetra-hydro-cannabinol (THC), or smoked. In addition, we should know the number of patients who regularly consumed stimulating substances such as coffee, cola, red bull, and other adrenergic drugs. Unless these stimulating substances are included in the evaluation, the results are not reliable.

We disagree with the notion in the introduction that neuropathic pain is generally associated with weakness, wasting, decreased tendon reflexes, allodynia, hyperalgesia, burning, tingling, and sudomotor dysfunction^1^. Neuropathic pain can occur without additional neurological symptoms or signs such as those occuring in small fiber neuropathy. Whether or not neuropathic pain is associated with additional neurological symptoms or signs depends largely on the underlying etiology of the neuropathic pain.

The finding that patients with radicular pain had less nocturnal pain than patients without radicular pain is contradictory to previous findings and requires a plausible explanation.

Overall, the interesting study has limitations that call into question the results and their interpretations. Clarifying these limitations would strengthen the conclusions and could add value to the study.
